# *QuickStats:* Percentage Distribution of Deaths Involving Injuries from Recreational and Nonrecreational Use of Watercraft,* by Month — United States, 2020–2022

**DOI:** 10.15585/mmwr.mm7319a5

**Published:** 2024-05-16

**Figure:**
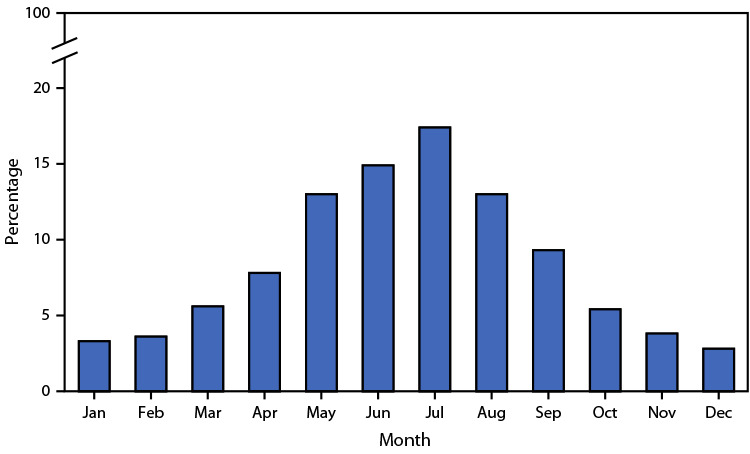
During 2020–2022, a total of 1,481 deaths occurred involving injuries from recreational and nonrecreational use of watercraft. The highest percentage of these deaths (17.4%) occurred in July, with the majority occurring during May–September.

**Supplementary Table:** https://stacks.cdc.gov/view/cdc/155045

**Source:** National Center for Health Statistics, National Vital Statistics System, Mortality Data, 2020–2022. https://www.cdc.gov/nchs/nvss/deaths.htm

For more information on this topic, CDC recommends the following link: https://www.cdc.gov/drowning/prevention/index.html

